# An Explainable Multi-Scale Deep Learning Framework for Multi-Class Brain MRI Classification

**DOI:** 10.3390/diagnostics16121791

**Published:** 2026-06-10

**Authors:** Hamoud H. Alshammari, Mahmood A. Mahmood

**Affiliations:** Department of Information Systems, College of Computer and Information Sciences, Jouf University, Sakaka 72441, Saudi Arabia; mamahmood@ju.edu.sa

**Keywords:** brain MRI classification, multi-class neurological diagnosis, EfficientNetV2-S, gated feature fusion, Grad-CAM explainability

## Abstract

**Background/Objectives:** Brain magnetic resonance imaging (MRI) is an important imaging modality for assessing neurological disorders. However, automatic multi-class MRI classification remains challenging because of visual similarity between disease categories, heterogeneous pathological patterns, class imbalance, and the need for reliable confidence estimation. This study aims to develop a comprehensive and well-calibrated deep learning framework for image-level brain MRI classification across multiple neurological categories. **Methods:** This paper introduces a new deep learning framework, MCND-ComputeNet++, for brain MRI classification into eight image-level categories using the MCND dataset, which comprises 16,400 two-dimensional brain MRI images belonging to eight diagnostic categories: AD-MildDemented, AD-ModerateDemented, AD-VeryMildDemented, BT-glioma, BT-meningioma, BT-pituitary, MS, and Normal. The proposed model uses a single pretrained EfficientNetV2-S backbone to extract hierarchical feature maps from three intermediate stages. These multi-level features are projected into a common latent space, spatially aligned, adaptively fused through learnable gated multi-scale fusion, further refined using convolutional processing, and aggregated using spatial attention pooling before classification. The training strategy combines class-balanced focal loss with label smoothing, MixUp/CutMix regularization, exponential moving average weight smoothing, warmup cosine learning-rate scheduling, temperature scaling, and test-time augmentation to improve generalization and calibration. The framework was evaluated using accuracy, precision, recall, macro-F1, macro-AUC, macro-average precision, expected calibration error, Brier score, bootstrap confidence intervals, ablation analysis, McNemar testing, and comparisons against standard pretrained baseline models. **Results:** MCND-ComputeNet++ achieved mean accuracy, macro-F1, macro-AUC, and macro-average precision values of 0.9738, 0.9771, 0.9993, and 0.9971, respectively, with narrow bootstrap confidence intervals indicating stable image-level performance. These findings should be interpreted as image-level/slice-level performance on MCND, because patient-level identifiers and subject-wise splitting were not available. These results outperformed most evaluated baselines, including ResNet50, DenseNet121, EfficientNetB0, EfficientNetV2-S with a standard classifier, Swin-Tiny, and ConvNeXt-Tiny, across several discrimination and calibration metrics. Compared with ConvNeXt-Tiny, the proposed model achieved higher macro-AUC and macro-average precision, together with a lower ECE and Brier score, suggesting improved image-level discrimination and confidence reliability. Compared with the EfficientNetV2-S standard classifier, accuracy increased from 0.9308 to 0.9738, while the Brier score decreased from 0.1045 to 0.0400. **Conclusions:** The results suggest that MCND-ComputeNet++ is a promising image-level brain MRI classification framework for the eight MCND categories. The proposed model integrates hierarchical feature extraction, shared latent projection, gated multi-scale fusion, convolutional refinement, spatial attention pooling, and calibrated inference within a unified architecture. However, because the current evaluation was conducted at the image/slice level without available patient-level identifiers, the findings should not be interpreted as patient-level clinical diagnostic validation. Further studies using subject-wise splitting, external multi-center datasets, 3D volumetric modeling, and multimodal clinical information are required to assess generalizability and potential clinical decision-support applicability.

## 1. Introduction

One of the most significant non-invasive imaging modalities used to assess the presence of neurological disorders due to the availability of abundant anatomical and pathological details without subjecting the patients to ionizing radiation is brain magnetic resonance imaging (MRI) [1,2]. MRI has become a focal point in clinical practice in the diagnosis and monitoring of multiple sclerosis, brain tumors, degenerative disorders, and other structural abnormalities of the brain [3,4]. Although MRI interpretation offers diagnostic value, interpretation is still time-consuming, and inter-reader variability exists, especially when the patterns of various disease classes have visual similarity or when small abnormalities need to be separated out from normal anatomical variation [5,6,7]. These issues have driven the growing use of deep learning in automated analysis of MRI, as convolutional neural networks (CNNs) can learn hierarchical image representations directly from the data and can be more effective than handcrafted feature-based pipelines in more challenging visual recognition problems [8].

Automated classification is one of the important applications of deep learning in medical imaging, as timely and accurate image-level identification of neurological patterns may support screening, triage, follow-up, and future decision-support research after patient-level validation. Recent studies have reported encouraging results in brain tumor subtype classification, Alzheimer’s disease staging, and MRI-based multiple sclerosis analysis using transfer learning and modern CNN backbones. Transfer learning is particularly important in brain MRI analysis because labeled medical imaging datasets are often much smaller than large-scale natural image benchmarks. In this context, pretrained models can provide strong low-level and mid-level visual representations that can be adapted to domain-specific imaging patterns. Consequently, ResNet, DenseNet, EfficientNet, EfficientNetV2, and ReXNet architectures have been widely used because of their favorable trade-offs among representational capacity, optimization stability, and computational efficiency [9,10,11,12,13].

Despite the importance of these developments, there are multiple shortcomings of the existing literature. To start with, much of the published literature is disease-specific, either on brain tumor classification, or Alzheimer disease recognition, or the analysis of multiple sclerosis lesions, but not in a multi-class neurological context. Second, most studies only compare a single or two different backbones with a single train/test split, and it is not always clear whether the reported gains are due to the architecture or are due to preferable experimental conditions. Third, aspects of reliability like confidence calibration and explainability are frequently considered auxiliary features of the learning pipeline, although clinically useful systems must deliver not just the correct prediction but also reliable confidence estimates and interpretable spatial evidence.

These gaps imply that a more detailed computational framework for heterogeneous brain MRI classification is required. Not only should such a framework perform well on predictive tasks, but it should also utilize hierarchical visual data better, be robust in terms of optimization, and have an easily interpretable prediction behavior. Based on this requirement, the current research paper introduces MCND-ComputeNet++, a multi-scale gated fusion classifier for classifying multi-class neurological brain MRIs. The offered framework applies a pretrained EfficientNetV2-S encoder to obtain hierarchical features across various backbone stages, projecting them into a common latent space and fusing them with adaptive gated fusion. The fused representation is further refined by attention-based feature aggregation and fed to a classification head where final decision-generating is performed. Moreover, to enhance the generalization, confidence reliability, and interpretability, the framework adds class-balanced focal loss, MixUp/CutMix regularization, exponential moving average (EMA), temperature scaling, test-time augmentation (TTA), and explainability using Grad-CAM.

The primary contribution of the work is thus not the comparison of pretrained backbones but, instead, the creation of a full and reliability-conscious MRI classification pipeline. Precisely, the research provides (i) a multi-scale hierarchical feature learning framework for heterogeneous neurological MRI classification; (ii) adaptive gated fusion to combine the information of shallow, mid-level, and deep encoder stages; (iii) attention-based aggregation, which allows greater strength of the spatial discriminativeness; and (iv) a confidence-aware optimization and calibration. With this design, MCND-ComputeNet++ will be used to offer a more balanced brain MRI classification solution that has strong discrimination, stable optimization, and reliable confidence estimation, and is interpretable in a clinically meaningful way.

The novelty of this paper is not limited to the single application of a pretrained CNN, calibration, or Grad-CAM, as these concepts have already been investigated in medical imaging. Instead, the hierarchical integration of EfficientNetV2-S feature maps via a multi-scale gated fusion mechanism and attention-based aggregation is the major contribution for the classification of heterogeneous eight-class brain MRI. The framework is then tested with calibration metrics, bootstrap confidence intervals, McNemar testing, ablation experiments, and visualization using Grad-CAM. This offers a more comprehensive image-level evaluation of a mixed neurological label space but also recognizes the need for external and patient-level validation.

## 2. Literature Review

Medical image analysis has fundamentally been transformed by deep learning, which has substituted manual feature engineering with end-to-end representation learning [5,6]. Surveys on a grand scale are always revealing that CNN-based pipelines are leading classification, segmentation, detection, and prognosis problems in radiology, pathology, and neuroimaging due to their capability to learn task-relevant features right from the data [5,6]. Transfer learning has gained relevance in the analysis of brain MRI, as visual encoders that are trained can be used to correct the small size of a dataset, as well as to enhance optimization behavior in downstream classification tasks. A systematic review of transfer learning in magnetic resonance brain imaging revealed that brain tumor analysis and dementia-related classification are some of the most common ones, and CNN-based backbones are the most widely used family of models [7].

The MRI classification backbone architectures employed in the modern world vary significantly in their information propagation and reuse. ResNet added residual learning to stabilize deeper network optimization [12], and DenseNet better utilized features by means of dense connectivity, frequently boosting gradient flow and parameter efficiency [11]. EfficientNet also introduced depth, width, and resolution scaling to obtain better accuracy–efficiency trade-offs [9], and EfficientNetV2 also optimized the speed of training and parameter efficiency [10]. ReXNet solved representational bottlenecks of lightweight convolutional networks by expanding channels gradually [13]. These design variations are of great concern in neuroimaging, where fine inter-class differences and similar patterns of disease demand both high representational sensitivity and consistency in optimization.

Besides CNN transfer learning, transformer-based models have gained a new relevance in MRI classification due to their capability to capture long-range spatial relationships. In neuroimaging applications where disease patterns are spread out, the Vision Transformer and Swin Transformer models offer the ability to capture more global anatomic relationships than just local convolutions. But these models typically need more training data or more powerful pretraining to prevent overfitting and may have widely differing results across different image resolutions, augmentation methods, and target datasets. Hence, comparisons of the accuracies of transformer-based MRI studies are not the only aspect of a study that should be considered, but also the size of the study, the number of labels in the study, the validation protocol used, and the computational costs.

One of the most widely investigated tasks in brain MRI deep learning is brain tumor classification, as different tumor subtypes may exhibit both shared and subtype-specific visual characteristics. Recent reviews of deep learning approaches for brain tumor MRI analysis have reported substantial progress in classification and segmentation; however, challenges related to generalization, dataset bias, and explainability remain unresolved [14]. Several transfer learning studies have shown that pretrained CNN architectures can achieve strong tumor-subtyping performance when combined with appropriate data augmentation and fine-tuning strategies [15,16,17,18]. Islam et al. reported a weighted accuracy of 98.73% and a weighted F1-score of 95.29% using fine-tuned transfer learning models [15], while Priyadarshini et al. achieved 96.20% accuracy using an EfficientNet-based architecture [16]; Ishaq et al. reported 98.60% accuracy using an enhanced EfficientNet-based model for four-class brain tumor classification [18]. Although these studies demonstrate the effectiveness of deep learning for tumor-specific MRI classification, they mainly focus on limited tumor categories and do not fully address broader neurological classification scenarios involving heterogeneous disease groups, such as Alzheimer’s disease, multiple sclerosis, normal cases, and multiple brain tumor subtypes within a unified framework [15,18].

There are also different methods of explainability, depending on what kind of evidence they provide. Since CNN-based architectures are used to generate class-discriminative heat maps from convolutional feature maps, Grad-CAM is applicable to them. LIME explains predictions by perturbing image regions and estimating local surrogate explanations, while SHAP estimates feature contributions by a game-theoretic formulation. While complementary interpretability can be obtained from both SHAP and LIME, they are both costly to compute for high-resolution medical images and can generate unstable explanations, depending on the segmentation or perturbation approach. For this reason, in the present study, the goal was to use Grad-CAM as the key visual explanation tool; however, future work is needed to compare Grad-CAM with SHAP- or LIME-based explanations to gain a fuller understanding of model behavior.

A further area of interest is structural MRI classification of Alzheimer’s disease (AD), where deep learning models are trying to pick up subtle changes in anatomy related to the advancement of the disease. The more recent studies and reviews show that CNNs, hybrid deep networks, and transfer learning approaches can be used to achieve high performance in the classification of Alzheimer’s disease versus control and in multi-stage severity recognition [19,20,21,22,23]. Vinukonda et al. achieved 98.12% accuracy and class-wise AUC values of 0.97 with moderate dementia [19], and Sorour et al. with CNN-LSTM showed good results with both 2D and 3D MRI-based AD classification, with the highest results of 99.92 in one compared setup [21]. Nevertheless, studies specific to Alzheimer’s are typically based on disease-specific data and relatively small label spaces [19,20,21,22,23]. Thus, good results in AD-only conditions do not always carry over to mixed neurological conditions where tumor, demyelinating, degenerative, and normal classes have to be separated at the same time.

Deep learning has also been of great benefit in multiple sclerosis (MS) research, particularly in lesion segmentation. Empirical experiments and reviews indicate that 3D CNNs, U-Net variants, attention-based networks, and multimodal networks have significantly enhanced lesion localization and, in others, downstream disease prediction [24,25,26,27,28]. A Dice similarity coefficient of 82.30% was reported by Hashemi et al. when using a modified attention U-Net to segment MS lesions [25], whereas Huang et al. used a joint segmentation–classification model to segment MS and NMOSD lesions and achieved a Dice of 74.87% with a classification performance of 92.36% [26]. Meta-suggestions and meta-analytic data also indicate that deep learning of MRI has a high diagnostic potential in MS, but there is high heterogeneity in datasets, imaging protocols, and modalities [28]. Although these advances have been made, the MS literature remains predominantly focused on lesion segmentation, as opposed to generalized image-level multi-class classification, and as a result is not directly applicable to larger neurological classification pipelines.

Simultaneously with raw predictive performance, explainability has become a key need in medical imaging, as often clinicians demand spatial accountability of automated predictions. One of the most popular visualization methods to apply to CNN-based models is Grad-CAM since it highlights areas of the image that have the most significant contribution to a target decision [29]. A recent survey of explainable AI in medical imaging highlighted that saliency-based descriptions are especially appealing in image-based diagnosis, but their clinical usefulness should be viewed with caution [30,31]. Explainability has also been employed in brain MRI in both tumor models and Alzheimer-related models to determine whether networks capture clinically relevant brain regions, as opposed to irrelevant artifacts [32,33]. Srinivas et al. integrated CNN-based brain tumor classification with Grad-CAM and achieved a maximum model accuracy of 95.86% with precision, recall, and F1-scores of approximately 0.95 [34].

One similar problem is that of calibration of confidence. It is possible that neural networks can be highly accurate and yet generate poorly calibrated probabilities, which can be an issue in medical decision support. One of the most commonly used post hoc calibration techniques is temperature scaling, as it is easy and useful in correcting probability overconfidence without altering the rankings of classes [35]. Moreover, label smoothing, MixUp, CutMix, and EMA-style weight averaging are regularization methods that have been demonstrated to enhance generalization, uncertainty behavior, and localization robustness in the classification of images [36,37]. These techniques prove to be highly applicable in studies of brain MRI since the clinically useful systems should be accurate and should also be stable, interpretable, and reliable in their confidence estimates.

A critical role of calibration in medical image classification is to ensure that predicted probabilities are clinically meaningful, not merely that the predicted class labels are correct. A model can be right on many samples and wrong on hard or elusive samples. A straightforward post hoc calibration procedure that modifies the shape of a probability distribution without altering the ordering of the predicted classes is called “temperature scaling.” Evaluation of the calibration should be done using both numerical statistics and visual reliability diagrams to detect confidence ranges where the model is overconfident or underconfident. Thus, calibration is not reported as an outcome metric but as part of the reliability assessment procedure.

In general, the literature shows that brain MRI deep learning has a high maturity, but there are still three critical gaps. First, the majority of studies remain disease-specific, and relatively less literature assesses models in a heterogeneous neurological label space. Second, architectural comparisons are usually carried out in a non-united environment, and it becomes hard to extrapolate the findings regarding the best backbone. Third, reliability-focused elements like calibration, TTA, explainability, and robustness analysis are not routinely included in comparative MRI classification pipelines as compared to raw accuracy measures. Such gaps are the primary reasons why the proposed MCND-ComputeNet++ framework is relevant, as it aims to classify multi-class neurological MRIs with a single architecture, based on multi-scale hierarchical feature extraction, gated fusion, attention-based aggregation, calibrated inference, and explainability-aware evaluation.

Many previous classification studies based on MRI have generated high-accuracy results in limited experimental conditions, such as binary tumor detection, four-class tumor subtype classification, and/or Alzheimer’s disease staging tasks alone. They are also very different tasks from heterogeneous neurological classification, in that they differ in label space, size of available datasets, imaging protocol, and degree of classification overlap. So it is important to consider the accuracy of the results without making a direct comparison. A more informative comparison should be made considering whether the model was tested on a single disease or multiple neurological classes; if it was tested on external data; if it was evaluated for calibration and interpretability; and whether the validation was done at the image level or at the subject level. In the present study, we bridge this gap by examining this unified task with eight classes using classification, calibration, ablation, confidence intervals, and explainability in an MRI dataset while acknowledging the need for external validation.

## 3. Proposed Approach

**Figure 1:**
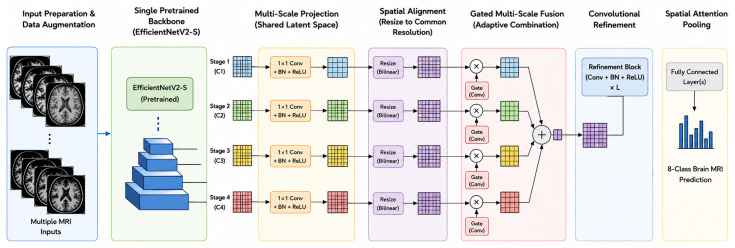
Architecture of the proposed MCND-ComputeNet++ framework.

The proposed MCND-ComputeNet++ is an image-level multi-class brain MRI classification framework for neurological category prediction. The model is based on a single pretrained EfficientNetV2-S backbone to extract hierarchical feature maps from several intermediate layers as shown in Figure 1. The proposed architecture not only allows preserving multi-level representations but also adaptively fuses them by using a gated multi-scale fusion mechanism instead of relying on the final backbone output. The advantage of this design is that the model can make use of complementary visual information such as local anatomical textures, intermediate structural patterns, and high-level disease-related semantic features.

The overall pipeline starts with input MRI preparation and augmentation. During training, resizing, tensor normalization, and spatial and appearance transformations are applied to each image. The augmented image is then fed into a pretrained EfficientNetV2-S encoder, which is used as a feature extractor. Selected backbone stages provide three intermediate feature maps. These maps are projected into a common latent representation using simple convolutional projection layers, resized to a common resolution, and then adaptively fused by learnable softmax gates. The fused feature map is further processed by convolutional refinement to enhance local consistency and reduce noisy activations. Finally, the most informative regions in the image are pooled into a compact feature vector, which is passed to a fully connected classification head for the final prediction of the eight brain MRI classes.

Besides its architectural design, MCND-ComputeNet++ includes several training and evaluation techniques to enhance robustness and reliability. The model is optimized using class-balanced focal loss, which reduces the negative effect of class imbalance and hard samples. The exponential moving average (EMA) of model weights is also used, and two mixed-sample regularization techniques, namely MixUp and CutMix, are applied during training. In addition, warmup and cosine annealing learning-rate schedules are incorporated, while temperature scaling and test-time augmentation are used during final prediction. The experimental design also includes ablation studies by removing gated fusion, attention pooling, EMA, mixed-sample augmentation, and calibration, together with baseline comparisons against standard CNN- and transformer-based models.

### 3.1. Input Preparation and Data Augmentation

MCND-ComputeNet++ first prepares raw MRI images for deep feature learning. The images are read from the dataset folder and converted to RGB format, which is the expected input format for ImageNet-pretrained models, before being resized to a fixed spatial resolution of 224×224. The pixel values are then converted into tensors and normalized using the ImageNet mean and standard deviation. This normalization enables the EfficientNetV2-S backbone to be effectively reused because it was originally pretrained under similar normalization settings.

During the training phase as shown in Algorithm 1, multiple data augmentation operations are applied to enhance the model’s ability to generalize. These include random cropping, random resizing, limited vertical flipping, rotation, and slight brightness and contrast perturbations. Such transformations help reduce overfitting by encouraging the model to learn disease-related patterns rather than memorizing fixed image positions or dataset-specific intensity distributions. In addition, the training pipeline probabilistically applies MixUp and CutMix. MixUp linearly interpolates between two images and their labels, whereas CutMix replaces a random patch of one image with a patch from another image. These regularization techniques smooth decision boundaries, reduce overconfidence, and improve robustness to class imbalance.
**Algorithm 1** Input preparation and data augmentation**Require:** Raw MRI image *I* and label *y***Ensure:** Augmented tensor $\tilde{I}$ and training label representation 1:Load the MRI image *I* from the dataset path. 2:Convert the image to RGB format. 3:Resize the image to 224×224. 4:During training, apply random resized crop, horizontal flip, limited vertical flip, rotation, and brightness/contrast jitter. 5:Convert the image into a tensor. 6:Normalize the tensor using ImageNet mean and standard deviation. 7:With probability *p*, apply MixUp or CutMix during mini-batch training. 8:Return the processed image tensor and the corresponding label.

### 3.2. Hierarchical Feature Extraction Using EfficientNetV2-S

The proposed model uses a single pretrained EfficientNetV2-S backbone instead of several parallel backbones. The backbone is implemented using the timm library with features_only=True, which enables the network to output intermediate feature maps instead of only the final classification representation. Specifically, three feature maps from selected intermediate stages are extracted using out_indices=(2,3,4). These maps are denoted as $F_{3}$, $F_{4}$, and $F_{5}$.

The hierarchical feature extraction strategy is important because different backbone depths capture different levels of information. The earlier selected feature map represents relatively finer anatomical and texture-related information, the intermediate feature map captures more structured morphological patterns, and the deepest feature map contains high-level semantic information related to the disease category as shown in Algorithm 2. By preserving these three feature levels, MCND-ComputeNet++ does not rely exclusively on the final deep representation but instead constructs a multi-scale description of each MRI image.

EfficientNetV2-S was chosen as the main encoder because it provides a favorable balance between capacity, efficiency, and training stability. It achieves strong high-level feature extraction, which is essential for 2D MRI classification in limited medical imaging scenarios, while reducing computational cost compared with heavier CNN architectures. EfficientNetV2-S also maintains a strong local inductive bias, which is useful for identifying local tissue boundaries, tumor regions, ventricular changes, and lesion-like patterns. Therefore, intermediate features from several stages are used to support the proposed multi-scale fusion design.
**Algorithm 2** Hierarchical feature extraction**Require:** Preprocessed MRI tensor $\tilde{I}$**Ensure:** Multi-level feature maps $F_{3}$, $F_{4}$, and $F_{5}$ 1:Feed $\tilde{I}$ into the pretrained EfficientNetV2-S encoder. 2:Forward propagate through the backbone layers. 3:Extract three intermediate feature maps from selected stages: 4:$F_{3}$: lower/intermediate representation. 5:$F_{4}$: deeper intermediate representation. 6:$F_{5}$: high-level semantic representation. 7:Preserve the three maps for projection, alignment, and fusion. 8:Return $F_{3}$, $F_{4}$, and $F_{5}$.

### 3.3. Shared Latent Projection

Each of the three extracted feature maps comes from a different layer in the EfficientNetV2-S encoder and therefore has a different channel dimension. As a result, they cannot be directly fused. To address this issue, MCND-ComputeNet++ uses a separate projection block for each feature map. Each projection block consists of 1×1 convolution, batch normalization, and SiLU activation. Through this operation, all feature maps are mapped into the same embedding dimension, which is set to 256 in the implementation.

The shared latent projection stage has two main roles as shown in Algorithm 3. First, it normalizes the channel dimension of all hierarchical features so that they can be combined in later stages. Second, it learns transformations that preserve useful information from each feature level while reducing representation mismatch between shallow, intermediate, and deep features.

(1) $$P_{3}=\phi_{3}\left(F_{3}\right),$$

(2) $$P_{4}=\phi_{4}\left(F_{4}\right),$$

(3) $$P_{5}=\phi_{5}\left(F_{5}\right),$$

where $\phi_{i}\left(\cdot \right)$ denotes the projection function applied to the corresponding feature map.
**Algorithm 3** Shared latent projection**Require:** Feature maps $F_{3}$, $F_{4}$, and $F_{5}$**Ensure:** Projected feature maps $P_{3}$, $P_{4}$, and $P_{5}$ 1:Apply a 1×1 convolutional projection to $F_{3}$: $P_{3}=\phi_{3}\left(F_{3}\right)$. 2:Apply a 1×1 convolutional projection to $F_{4}$: $P_{4}=\phi_{4}\left(F_{4}\right)$. 3:Apply a 1×1 convolutional projection to $F_{5}$: $P_{5}=\phi_{5}\left(F_{5}\right)$. 4:Each $\phi_{i}$ consists of 1×1 convolution, batch normalization, and SiLU activation. 5:Return the projected feature maps $P_{3}$, $P_{4}$, and $P_{5}$.

### 3.4. Spatial Alignment and Gated Multi-Scale Fusion

After projection, the feature maps still have different spatial resolutions. Therefore, $P_{3}$ and $P_{4}$ are resized to match the spatial size of $P_{5}$, which is the deepest feature map. This alignment is performed using bilinear interpolation, resulting in feature maps with a common channel width and a common spatial resolution.

The aligned features are then adaptively fused using a learnable gated fusion mechanism. In this model as shown in Algorithm 4, the learnable gate vector contains three values, corresponding to the three feature levels. The contribution of each scale is learned during training by normalizing these gate values using a softmax function. The final mixed representation is obtained by computing a weighted summation of the aligned projected features. In the gated fusion ablation, this learnable weighted fusion is replaced with a simple average of the three feature maps. Therefore, the ablation directly evaluates the contribution of adaptive scale weighting to classification performance.

(4) $$\alpha_{i}=\frac{\exp(g_{i})}{\sum_{j=1}^{3}\exp\left(g_{j}\right)},$$

(5) $$F_{\mathrm{mix}}=\alpha_{1}P_{3}+\alpha_{2}P_{4}+\alpha_{3}P_{5}.$$

**Algorithm 4** Spatial alignment and gated fusion**Require:** Projected feature maps $P_{3}$, $P_{4}$, and $P_{5}$**Ensure:** Mixed multi-scale feature map $F_{\mathrm{mix}}$ 1:Select the spatial resolution of $P_{5}$ as the target resolution. 2:Resize $P_{3}$ to the spatial size of $P_{5}$. 3:Resize $P_{4}$ to the spatial size of $P_{5}$. 4:Define a learnable gate vector $g=[g_{1},g_{2},g_{3}]$. 5:Apply softmax normalization to obtain $\alpha_{1},\alpha_{2},\alpha_{3}$. 6:Fuse the aligned feature maps using:$$F_{\mathrm{mix}}=\alpha_{1}P_{3}+\alpha_{2}P_{4}+\alpha_{3}P_{5}.$$ 7:Return the fused multi-scale representation.

### 3.5. Convolutional Refinement

The fused feature map is then fed into a convolutional refinement module. This model as shown in Algorithm 5 is implemented as two consecutive convolutional blocks. Each block consists of a 3×3 convolution, batch normalization, and SiLU activation, with dropout applied between the two blocks. In this refinement phase, the local spatial consistency of the fused representation is enhanced, while noisy activations caused by multi-scale merging are reduced.

This stage is important because simple feature fusion can combine both useful and irrelevant activations from different backbone layers. After adaptive scale weighting, the convolutional refinement module further improves the fused representation. Consequently, the refined feature map becomes more discriminative before being passed to the spatial attention pooling module.
**Algorithm 5** Convolutional refinement**Require:** Mixed feature map $F_{\mathrm{mix}}$**Ensure:** Refined feature map $F_{\mathrm{ref}}$ 1:Apply a 3×3 convolutional block to $F_{\mathrm{mix}}$. 2:Apply dropout regularization to reduce overfitting. 3:Apply a second 3×3 convolutional block. 4:Each convolutional block consists of convolution, batch normalization, and SiLU activation. 5:Return the refined feature map $F_{\mathrm{ref}}$.

### 3.6. Spatial Attention Pooling

The feature map obtained after refinement still contains spatial activations. Since not all spatial regions contribute equally to neurological disorder classification, MCND-ComputeNet++ employs a spatial attention pooling module. In this module as shown in Algorithm 6, a single-channel attention map is predicted from the refined feature representation. The attention map is flattened across spatial positions and normalized using the softmax function so that the weights of all spatial positions sum to one.

The refined feature map is then spatially weighted and summed to form the final global feature vector for the image. Spatial attention pooling enables the model to focus on diagnostically informative regions and reduce the contribution of irrelevant background areas. In the ablation study, this module is removed and replaced by adaptive average pooling to measure the contribution of attention-based feature aggregation.

(6) $$z=\sum_{h=1}^{H}\sum_{w=1}^{W}A\left(h,w\right)F_{\mathrm{ref}}\left(:,h,w\right).$$

**Algorithm 6** Spatial attention pooling**Require:** Refined feature map $F_{\mathrm{ref}}\in R^{C\times H\times W}$**Ensure:** Global feature vector *z* 1:Feed $F_{\mathrm{ref}}$ into the spatial attention block. 2:Generate a single-channel attention map *A*. 3:Flatten *A* across all spatial positions. 4:Apply softmax normalization over the H×W positions. 5:Multiply the normalized attention map by $F_{\mathrm{ref}}$. 6:Sum the weighted features across spatial locations. 7:Return the global feature vector *z*.

### 3.7. Classification Head and Decision Generation

The global feature vector obtained from spatial attention pooling is fed into a fully connected classification head. The classifier consists of a linear layer, batch normalization, SiLU activation, dropout, and a final linear output layer. The output size of the final layer is equal to the number of MCND classes, which is eight in the proposed task. The raw logits are passed through the softmax function to obtain posterior class probabilities.

The predicted class is selected as the class with the highest posterior probability. The task is formulated as a single-label multi-class classification problem, where each MRI image belongs to one neurological disorder category. Therefore, the decision head is trained to produce mutually exclusive class probabilities across the eight categories.

(7) $$p_{i}=\frac{\exp(l_{i})}{\sum_{j=1}^{K}\exp\left(l_{j}\right)},$$

(8) $$\hat{y}=\arg\max_{i}p_{i}.$$

### 3.8. Class-Balanced Focal Optimization

The class distribution of the MCND classification task may be imbalanced, which can reduce the effectiveness of the standard cross-entropy loss function. To address this issue, the proposed model uses a class-balanced focal loss. The class-balanced term is scaled according to the effective number of samples in each class, giving greater weight to underrepresented classes. The focal term reduces the contribution of easy samples so that training focuses more on challenging or confusing samples.

The implementation also includes label smoothing, which prevents the model from becoming overly confident during training. In medical image classification, class boundaries can be subtle and disease patterns may overlap. Therefore, combining label smoothing with class-balanced focal loss promotes stable and balanced learning across the eight classes.

(9) $$w_{c}=\frac{1-\beta }{1-\beta^{n_{c}}},$$

(10) $$L=w_{y}{\left(1-p_{t}\right)}^{\gamma }CE\left(l,y\right).$$

### 3.9. Regularized Training with MixUp, CutMix, EMA, and Scheduled Learning

The training strategy is designed to improve generalization and optimization stability using multiple mechanisms. First, MixUp and CutMix are used probabilistically during training. These techniques produce mixed samples and mixed targets, leading to smoother decision boundaries and reduced memorization of class-specific artifacts. Second, the model is optimized using AdamW with weight decay to improve generalization. Third, gradient clipping is applied to avoid unstable gradient updates.

The learning rate is controlled using warmup and cosine annealing schedules. During the warmup phase, the learning rate is gradually increased to stabilize the fine-tuning of the pretrained backbone. After warmup, cosine decay gradually reduces the learning rate toward a minimum value. EMA is also employed to maintain a smoothed version of the model weights. The EMA model is used during validation and checkpoint selection, which can provide more stable performance than using only the instantaneous training weights.

(11) $$\theta_{\mathrm{EMA}}\leftarrow \lambda \theta_{\mathrm{EMA}}+\left(1-\lambda \right)\theta .$$

### 3.10. Calibration, Test-Time Augmentation, and Evaluation

The model applies post hoc temperature scaling after training to improve confidence calibration. Temperature scaling learns a scalar parameter from the validation logits and labels, and then divides the test logits by this scalar before applying softmax. This procedure adjusts the confidence values without changing the predicted class ranking. The calibrated probabilities are then used to compute performance and reliability indicators, including expected calibration error and Brier score.

The implementation also enables test-time augmentation. During inference, the model processes both the original image and a horizontally flipped version, and the logits from both predictions are averaged. This reduces sensitivity to small spatial variations and improves prediction consistency. Evaluation includes accuracy, macro-F1, micro-F1, macro-AUC, macro-average precision, confusion matrix, ROC curve, PR curve, reliability diagram, bootstrap confidence intervals, and McNemar statistical testing against the full model in the ablation setting.

### 3.11. Implementation Configuration

Table 1 shows the hyperparameter settings of the MCND-ComputeNet++ model and the compromise between learning stability, computational efficiency, and predictive performance. To enhance reproducibility, a constant random seed of 42 was used. The input image size was set to 224×224 to preserve important anatomical information while maintaining reasonable computational complexity. The model was trained with a batch size of 24 and two data-loading workers, and CUDA acceleration was used to optimize training. To obtain a more reliable estimate of generalization performance, experiments were conducted using five-fold cross-validation. The maximum number of training epochs was set to 35, with three warmup epochs to stabilize initial optimization and an early stopping patience of eight epochs to avoid overfitting when validation performance stopped improving. A dropout rate of 0.30 was used to reduce overfitting and enhance generalization. The initial learning rate was $2\times 10^{-4}$, the minimum learning rate was $1\times 10^{-6}$, and the weight decay was $1\times 10^{-4}$, all of which help stabilize parameter updates and improve regularization.

The input size of 224×224 was selected because it ensures compatibility with pretrained ImageNet encoders while keeping GPU memory usage under control during five-fold training. The batch size of 24 was selected as the largest stable value for the available computational environment. AdamW and a weight decay of $1\times 10^{-4}$ were used to improve fine-tuning stability and prevent overfitting. A classification head with a dropout rate of 0.30 was used for regularization in the final representation. Class-balanced focal loss was applied to reduce majority-class dominance and increase the contribution of hard samples. MixUp and CutMix were applied only during training to smooth decision boundaries, while EMA was used to stabilize the final model parameters. Temperature scaling was applied to validation predictions to improve probability calibration without affecting class rankings.

## 4. Experimental Results

This section summarizes in a structured way the experimental results obtained using the proposed MCND-ComputeNet++ framework. The characteristics of the MCND data are outlined, and the image-level evaluation protocol is presented. Next, the five-fold performance of the cross-validation is presented with fold-wise metrics, aggregated classification results, and summary statistics. Then, the model is evaluated in terms of its reliability through the bootstrap confidence intervals, the calibration metrics, and the reliability curves. Qualitative behavior of the model is also discussed with the help of the confusion matrix analysis, ROC and precision–recall curves, training curves, learning-rate behavior, and Grad-CAM visualizations. Finally, ablation studies, statistical comparisons, baseline model comparisons, and an evaluation of external datasets are reported to explain the contribution, stability, and generalization performance of the proposed framework.

### 4.1. Dataset

**Figure 2:**
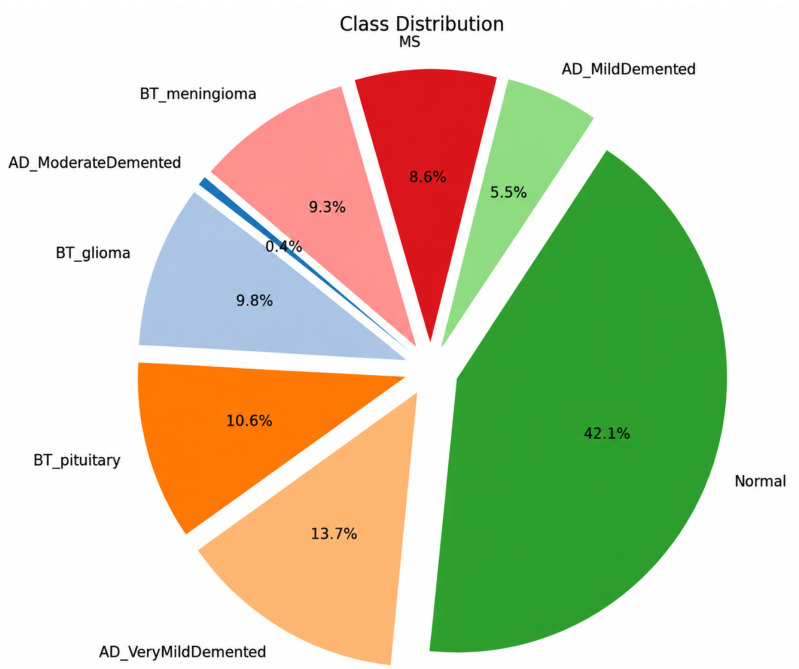
Dataset class distribution.

**Figure 3:**
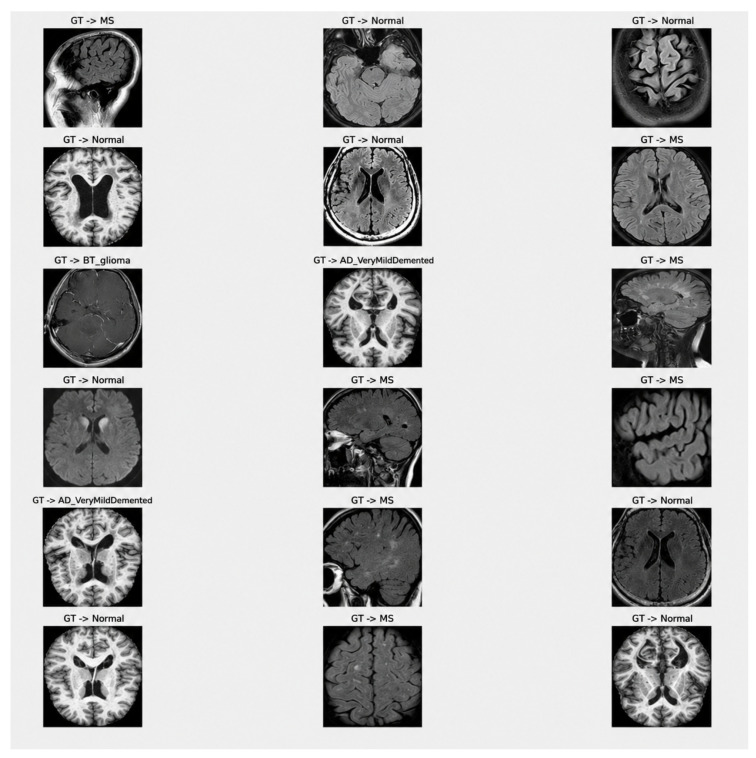
Dataset samples.

The MCND dataset was used in the experiments. It consists of 16,400 brain MRI images belonging to eight diagnostic categories: AD-MildDemented, AD-ModerateDemented, AD-VeryMildDemented, BT-glioma, BT-meningioma, BT-pituitary, MS, and normal. Figure 2 and Figure 3 show the class distribution and representative examples from each category, respectively. Since the dataset is provided with image-level diagnostic labels, this study formulates the task as an eight-class brain MRI image classification problem. In addition, because the dataset provides two-dimensional MRI slices rather than complete volumetric scans, the analysis was conducted as an image-based classification task rather than a 3D volumetric classification or lesion-based segmentation problem [38].

The proposed model was designed to process each MCND image slice as an independent MRI sample, as the dataset is presented in the form of 2D images rather than full volumetric MRI examinations. This design reduces computational complexity and enables the use of pretrained 2D image encoders. However, it does not model anatomical continuity across adjacent slices or capture the full volumetric extent of disease. In real-world radiological diagnosis, multiple slices, imaging sequences, anatomical context, and patient clinical history are often required. Therefore, the current framework should be interpreted as an image-level classification model and not as a comprehensive clinical diagnostic system.

The MCND dataset consists of image-level 2D MRI slices labeled with diagnostic categories. Patient-level identifiers were not available in the dataset metadata; therefore, stratified image-level splitting was used to preserve class proportions across the five cross-validation folds. This design provides an estimate of image-level classification performance, but it cannot guarantee complete subject-level separation if multiple images or correlated slices from the same subject are present in the dataset. Strict image-level separation was maintained between training and validation folds. However, because patient identifiers were unavailable, subject-level leakage or correlation between slices from the same subject cannot be fully excluded. Accordingly, the reported results should be interpreted as slice-level performance on the MCND dataset rather than patient-level diagnostic performance. This limitation was considered when interpreting the results, and future studies should use subject-wise splitting and external validation when patient-level metadata become available.

To obtain a more reliable estimate of model performance, the dataset was evaluated using five-fold stratified cross-validation rather than a single fixed train–validation–test split. In each fold, the data were divided to maintain the class proportions in both the training and validation subsets. All MRI images were resized to 224 × 224 pixels and preprocessed according to the input requirements of the pretrained network. To improve generalization and reduce overfitting, data augmentation was applied only to the training subset in each fold, while validation images were kept unchanged. Across all folds, strict image-level separation was maintained between the training and validation subsets. However, due to the absence of patient-level identifiers, the cross-validation results should be regarded as an objective assessment of image-level model performance, while possible subject-level correlation cannot be completely ruled out.

### 4.2. Results

Table 2 shows the cross-validation performance of the proposed MCND-ComputeNet++ framework when applied to five folds. The model demonstrated high accuracy values for all folds with values ranging from 0.9687 to 0.9769; this shows that the model has the ability to classify with the same accuracy across various validation partitions. Additionally, the macro-F1 scores were high, ranging between 0.9715 and 0.9822, indicating that the model performed well in all the healthy and unhealthy classes, with no particular bias towards the major classes. The discrimination metrics also reflect the robustness of the proposed model. macro-AUC was found between 0.9992 and 0.9994, and macro-average precision was between 0.9962 and 0.9975, indicating good one-vs-rest class separability and stable precision–recall performance on all folds. The micro and weighted metrics also aligned with the accuracy, indicating the model works well both at a global level and at a class-weighted level. After temperature scaling, the calibration metrics yielded good and reliable confidence estimates. The ECE values were found to be in the range of 0.0118–0.0190, and the Brier values were found to be in the range of 0.0320–0.0480, showing that the predicted probabilities were closer to the actual correctness of predictions. The narrow range of the accuracy, macro-F1, macro-AUC, and macro-AP bootstrap confidence intervals across folds emphasizes the model’s stability. Table 2 shows that overall, MCND-ComputeNet++ achieved good and consistent image-level performance across all five folds of cross-validation.

The classification report for the proposed model on the eight diagnostic classes is given in Table 3. The results of the model are good, with an accuracy of 0.9738, a macro-F1 score of 0.9771, and a weighted F1 score of 0.9738. The highest class-level performance was found for the brain tumor categories, especially BT_glioma, BT_meningioma, and BT_pituitary, with F1-scores of 0.9938, 0.9873, and 0.9925, respectively. This means that the model can distinguish the different types of tumors from one another effectively. The Alzheimer’s disease classes performed well as well, particularly “AD_MildDemented” and “AD_ModerateDemented,” with F1-scores of 0.9906 and 0.9771, respectively. The smaller precision and F1-scores for the AD_VeryMildDemented and MS classify because there are subtle differences in the visual appearance or perhaps overlap between these classes and other MRI patterns. The normal class had good precision, but a relatively low recall, meaning that in the normal class, there were some samples that were mistakenly labeled as patients. Overall, a high macro- and weighted average indicate that the proposed model yields the same performance level across all classes and has a balanced classification performance.

The cross-validation performance of the proposed model for all the folds is summarized in Table 4. The model performance was high, and the overall classification performance was good; the mean accuracy was 0.9738, and the macro-F1 was 0.9771. The low standard deviations for accuracy (0.0033), macro-F1 (0.0052), and weighted F1-score (0.0033) indicate the consistency of the model across folds. Excellent class discrimination and precision–recall behavior are evident in the very high macro-AUC (0.9993) and macro-AP (0.9971). Further, the ECE value of 0.0154 and Brier score of 0.0400 of the model suggest good calibration and reliable probability estimates. Overall, the cross-validation results validate the proposed model as accurate, stable, well-calibrated, and robust to various splits in data.

Table 5 gives the 95% confidence intervals of the evaluation metrics that are important in the main evaluation of the proposed model, and the statistical evidence of the reliability and stability of the overall performance of the model is provided. The accuracy interval of 0.9711–0.9760 reported implies that the model is highly accurate in its classification with a small margin of error. In the same vein, the macro-F1 confidence interval, 0.9727 to 0.9807, indicates that the model has a good and balanced performance across all classes instead of being overwhelmed by the bigger classes. The macro-AUC confidence interval of 0.9990–0.9994 indicates a very high degree of separability between the target classes and indicates that this ability to discriminate is very robust to resampling. Similarly, the macro-average precision (Macro-AP) range (0.9952 to 0.9971) represents a very high confidence in the precision–recall characteristic across classes. In general, the small values of all reported confidence intervals indicate that the proposed framework is not only correct but also statistically strong, with not much fluctuation in the performance estimates, contributing to the confidence in its generalization capabilities in multi-class brain MRI classification.

**Figure 4:**
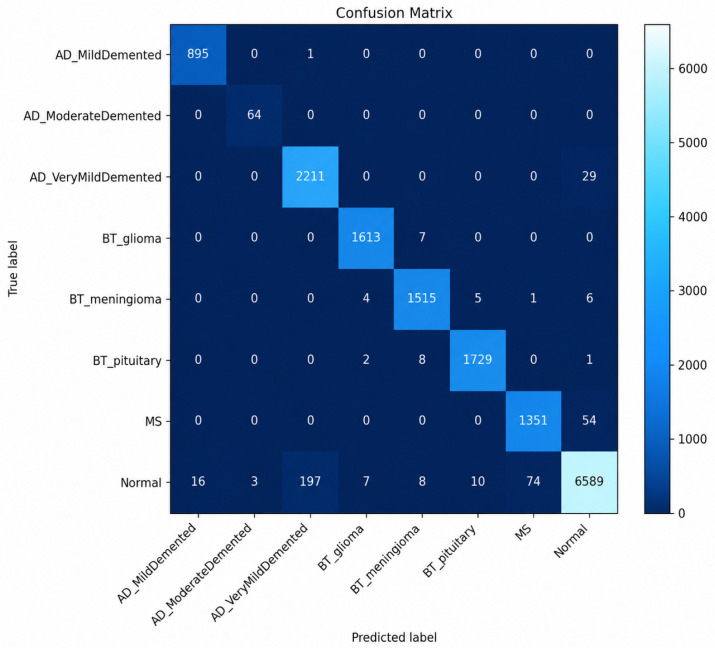
Confusion matrix of the proposed model.

The confusion matrix of the proposed model is shown in Figure 4 and reveals the model’s behavior in class-wise predictions on the eight neurological categories. The prevailing diagonal pattern implies that the model has very high correct classification rates on most classes, which corroborates good overall discrimination performance. Specifically, AD_MildDemented, AD_ModerateDemented, BT_glioma, BT_meningioma, BT_pituitary, and MS exhibit a high proportion of correctly classified samples with a small amount of off-diagonal confusion, indicating that these classes are well-separated in the learned feature space. The most pronounced mistakes are observed between AD_VeryMildDemented and normal as some very mild cases of dementia are forecasted as normal and vice versa. This trend is unsurprising due to the very mild dementia, which is likely to present subtly with structural changes that can even be physically intermingled with normal brain appearance. A similar but lesser level of confusion is also seen between certain tumor subtypes, especially BT glioma, BT meningioma, and BT pituitary, which show partial similarity in tumor-related MRI features. The MS class is also somewhat confused with normal, which could be explained by the heterogeneous and even inconspicuous radiological presentation of multiple sclerosis. In general, the figure shows that the suggested framework offers very precise multi-class classification and reveals that the primary residual problem is the difficulty in recognizing fine disease types, particularly very mild dementia and some normal or nearly related pathological patterns.

The primary ambiguity is between AD-VeryMildDemented and normal, which is theoretically reasonable since subtle differences in structure could be seen as a normal anatomical variation in individual 2D slices when very mild dementia occurs. There also is some overlap with normal, probably due to slice selection and lesion burden. However, BT-glioma, BT-meningioma, and BT-pituitary are more separable, as abnormalities caused by the tumor tend to be more spatially restricted and clearly visible. The class-wise patterns hint at the fact that the model is more successful when the abnormalities are conspicuous but fails more in subtle neurodegenerative and demyelinating cases.

**Figure 5:**
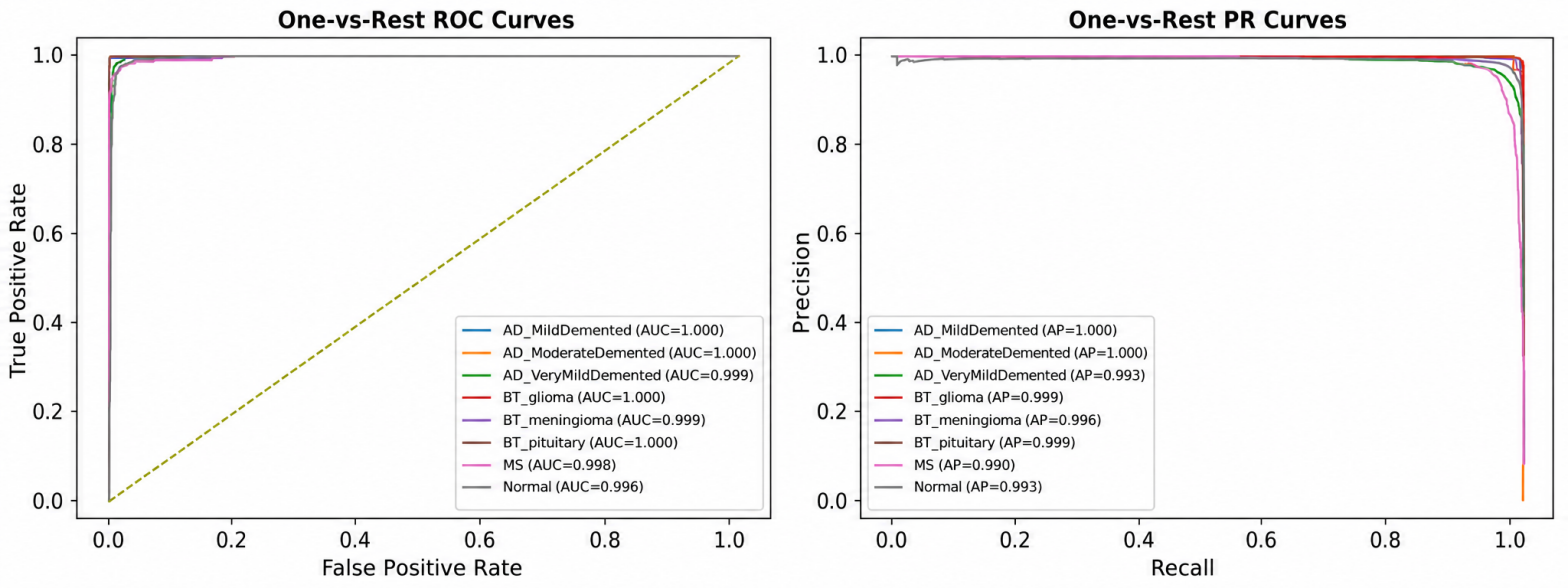
Roccurve and PR curve for proposed model.

Figure 5 shows the one-v-rest ROC and precision–recall (PR) curves of the proposed model and indicates that it has an excellent discriminative performance in all the neurological classes. All the curves of the classification in the ROC plot are drawn to the upper-left region and are much higher than the diagonal reference line, which means there are high true positive rates and low false positive rates. This is demonstrated by the almost perfect AUC values, which lie between 0.996 and 1.000, attesting to the fact that the model is highly effective in separating the target classes. Equally, the PR curves are found to be near the upper-right part of the plot, indicating that the model maintains a very high level of accuracy over a wide scale of recall values. The average precision (AP) values reported, with a range of 0.990 to 1.000, also confirm the reliability of the classifier during the class-imbalance-sensitive evaluation. The classes that have almost perfect ROC and PR behavior are AD_MildDemented, AD_ModerateDemented, BTglioma, and BTpituitary, which are slightly lower but still outstanding in comparison to MS, normal, and AD_VeryMildDemented, which are more difficult due to higher visual overlap with other classes. On the whole, the figure attests that the suggested framework delivers very high levels of class separability and robustness of the precision–recall performance, which supports the effectiveness of the framework in the context of multi-class brain MRI classification.

**Figure 6:**
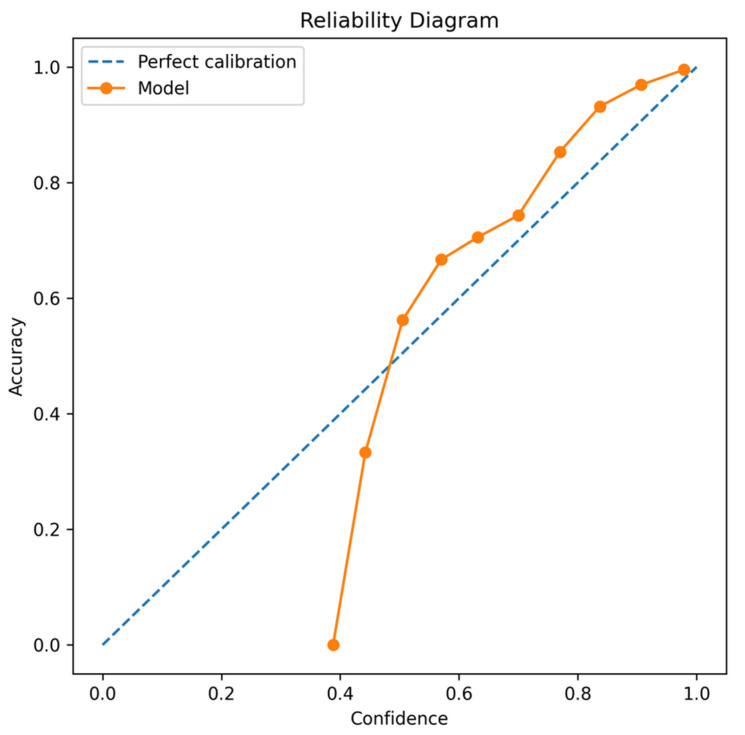
Reliability diagram for proposed model.

Figure 6 shows the reliability diagram of the proposed model and demonstrates the dependence between the predicted confidence and observed accuracy, thus giving a visual evaluation of the calibration of probabilities. The thin diagonal line indicates ideal calibration, where the confidence of the model perfectly reflects the actual likelihood of making a prediction that is correct, whereas the orange curve indicates the actual calibration behavior of the proposed framework. All in all, the model has a reasonably good calibration. At the mid-confidence range, there is a deviation from the ideal calibration line, which shows that the model is overconfident or underconfident at some points. However, the overall movement of the curve is upwards and near the reference line, with high confidence rates indicating that the probability estimates of the model are not highly inaccurate and clinically significant. These results are aligned with the comparatively low anticipated error in calibration as indicated in the quantitative results and suggest that the suggested framework not only achieves high classification performance but also generates confidence scores that are reliable enough to be used in medical decision-support settings.

The reliability diagram suggests satisfactory reliability, although this is not consistent over all confidence levels. The model is more likely to be accurate when the predicted accuracy is high, in the “high confidence” window. Some of the deviations in the mid-confidence range indicate that there might be some cases that are still overconfident or underconfident. In clinical decision support, these predictions between confidence levels should not be used in the same manner as highly confident predictions. A practical deployment strategy might help define confidence thresholds such that low- or mid-confidence cases would be sent for radiologist review and not accepted automatically. As such, calibration is not just a reporting metric; it is also a way to safer decision triage.

**Figure 7:**
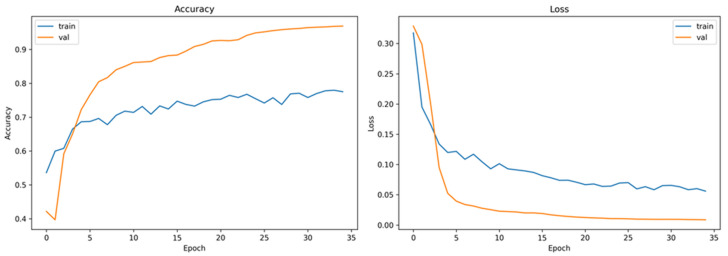
Accuracy and loss for proposed model.

In Figure 7, training and validation accuracy and loss curve plots of the proposed model during the training epochs are provided, which give a clear picture of how it converges and the stability of its learning process. In the accuracy plot, both curves increase gradually with training, and the validation accuracy increases quickly and stays at a consistently higher level than the training accuracy, eventually approaching a very high value towards the end of training. The training and validation losses in the loss plot decrease consistently; the validation loss decreases sharply in the initial epochs and then decreases further to a very low value, which means that effective optimization and good generalization behavior are achieved. The fact that both plots do not experience unstable oscillations or divergence indicates that the training process is under control, and the choice of the optimization strategy, regularization methods, and model design positively affects convergence. Though the validation curves are better behaved than the training curves, in general, the figure shows that the proposed framework learns effectively, converges smoothly, and achieves stable performance without any apparent signs of detrimental overfitting.

**Figure 8:**
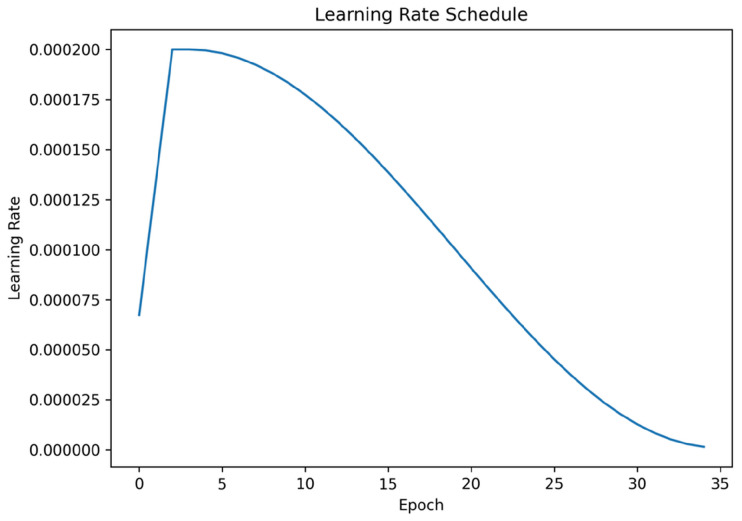
Learning rate curve for proposed model.

The learning-rate schedule used to train the proposed model is presented in Figure 8, with a warmup followed by a cosine decay strategy between the training epochs. The initial value of the learning rate is smaller than the ultimate learning rate, and during the initial few epochs, the learning rate rapidly increases to the optimal learning rate and stabilizes to avoid sharp updates when fine-tuning pretrained network parameters. Once this warmup period ends, the learning rate is reduced in a cosine annealing schedule, slowly shifting away from exploration and more towards refined optimization as training continues. The advantage of this schedule is that it enables the model to learn aggressively in the initial stages and carefully in the subsequent epochs. The steady decreasing pattern that appears in the figure means that there is a controlled optimization process without any abrupt changes, which leads to stable convergence and minimizes the risk of overshooting the optimal solutions, and which also leads to the high final classification performance of the proposed framework.

**Figure 9:**
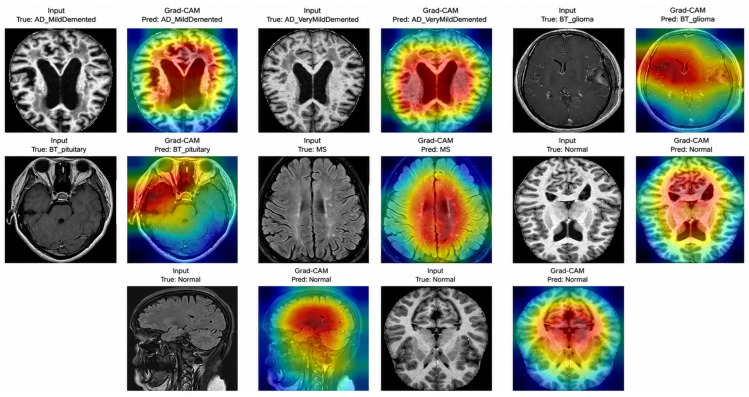
Grad CAM visualizations for different brain MRI classes, including AD MildDemented, AD VeryMildDemented, BT glioma, BT pituitary, MS, and Normal. For each sample, the original input MRI image is shown alongside its corresponding Grad-CAM heatmap. The heatmap highlights the image regions that contributed most strongly to the model’s prediction. Warm colors (red and yellow) indicate regions of highest importance and strongest model attention, green indicates moderate importance, whereas cool colors (blue) represent areas of low contribution or minimal influence on the classification decision.

Figure 9 shows examples of Grad-CAM visualizations of the proposed model and offers qualitative indicators of the spatial areas that played the biggest role in the final class predictions. The initial brain MRI image of the sample is displayed, and the respective Grad-CAM heatmap is presented as well, with warmer colors indicating a more significant impact on the decision. The findings indicate that the model tends to concentrate on the anatomically significant areas as opposed to unrelated background structures, which justifies the decipherability of the framework. The attention maps in the samples relating to Alzheimer’s are localized in the middle areas of the brain and the ventricular compartments, implying that the model is attentive to the patterns that are related to tissue atrophy and structural variations. The highlighted regions in the brain tumor examples are localized over abnormal mass-relevant areas, which suggests that the model captures lesion-relevant features to discriminate tumor subtypes. In the multiple sclerosis sample, active regions highlighted by the activation map include central and periventricular areas, which are frequently of clinical interest in the demyelinating disease. In normal cases, the focus of attention is more diffusely spread out across stable anatomical structures, which signifies the lack of local pathological abnormalities. All in all, the figure shows that the proposed model not only performs well in terms of quantitative performance but also grounds its decisions on visually plausible brain areas, which enhance the clarity of its transparency and suitability to medical decision-support systems.

### 4.3. Ablation Study

In order to measure the contribution of each component in the proposed framework, an ablation study was carried out, which is presented in Table 6. The full model achieved the best overall performance, with the highest accuracy (0.9738), macro-F1 (0.9771), macro-AUC (0.9993), and macro-AP (0.9971), as well as the lowest ECE (0.0154) and Brier score (0.0400). This suggests that the overall system has the potential to classify a sufficient number of images and offer dependable probability estimates. Single-component removal had various performance decrement effects. The no_attn_pool variant resulted in the lowest accuracy (0.9375) and macro-F1 (0.9399), highlighting the need for attention-based pooling for discriminative feature aggregation. Calibration is particularly important for accurate confidence estimation, as removing it (no_calibration) led to the biggest degradation in the ECE (0.2213) and Brier score (0.1556). The no_mixing variant and the variants no_gated_fusion and no_ema also demonstrated lower accuracy and macro-F1 and higher calibration error than the full model. From an overall perspective, the results validate the enhanced robustness, discrimination capacity, and reliability of the proposed model by combining the advantages of data mixing, calibration, gated fusion, EMA stabilization, and attention pooling.

The statistical comparison of each ablation variant and the full model is shown in Table 7. Performance differences were calculated as ablation variant minus full model; therefore, negative values indicate that the full model performed better. For all the ablation variants, the accuracy and macro-F1 decreased, which verified that the removal of any significant component affected the overall performance. The largest error seen was with no_attn_pool, no_ema, no_gated_fusion, no_calibration, and no_mixing. For the macro-F1 indicator, the greatest decrease was seen in no_attn_pool, and no_calibration had a significant decrease. The McNemar test and bootstrapped differences remained statistically significant for the no_mixing variant, indicating that the model generalization was improved by MixUp/CutMix regularization. Overall, statistical analysis confirms the contribution of the suggested components to the robustness of the classification, the balance of classes, and the reliability of probabilities.

**Figure 10:**
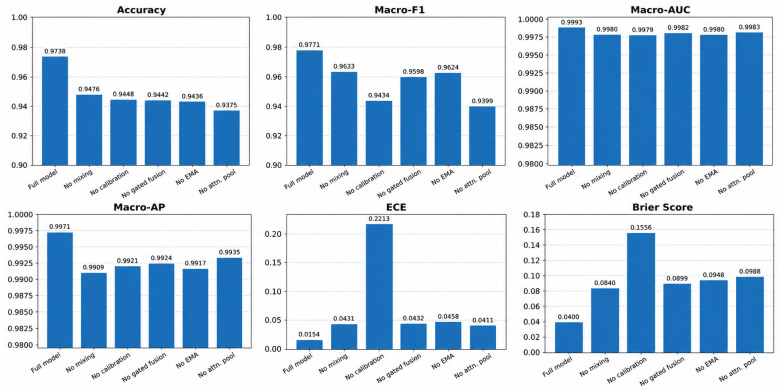
Ablation study of proposed framework.

The results of the ablation study to check the contribution of each component in the proposed framework are summarized in Figure 10. The results show that the full model exhibited the highest overall classification, discrimination, and calibration performance. The no_mixing variant is employed to assess whether MixUp/CutMix regularization is required for improving generalization and preventing overfitting. Similarly, the performance of the no_gated_fusion, no_ema, and no_attn_pool variants is competitive but not consistent in comparison to the full model in terms of discrimination metrics such as macro-AUC and mAP. The results indicate that the classification and the prediction reliability decrease when one of the main components is deleted. In general, the ablation results show that the proposed data mixing regularization, gated multi-scale fusion, EMA-based training stabilization, attention-based pooling, and calibration have a synergic effect on the robustness of the final architecture and its reliability.

### 4.4. Baseline Comparison

The proposed MCND-ComputeNet++ is compared with standard pretrained models for multi-class brain MRI classification in Table 8. The proposed model achieved the best overall performance, with an accuracy of 0.9738, macro-F1 of 0.9771, macro-AUC of 0.9993, macro-AP of 0.9971, ECE of 0.0154, and Brier score of 0.0400. When compared with the best baseline ConvNeXt-Tiny, MCND-ComputeNet++ achieved an increase in accuracy from 0.9421 to 0.9738 and a decrease in ECE from 0.0367 to 0.0154, for better classification performance and more reliable confidence estimation. Other baselines like DenseNet121, EfficientNetV2-S, EfficientNetB0, Swin-Tiny, and ResNet50 recorded lower performance, particularly for accuracy, macro-F1, and Brier score. In summary, the results indicate that the proposed framework can offer excellent prediction performance and reliability of calibration for brain MRI classification.

### 4.5. Comparison with Other Dataset

#### Related-Task Evaluation on an External Four-Class Brain Tumor MRI Dataset

The related-task evaluation brain tumor MRI dataset contains 7200 human brain MRI images distributed across four well-balanced classes: glioma, meningioma, pituitary tumor, and normal, also referred to as no tumor. The dataset is organized into training and testing subsets and is designed to support medical image analysis and deep learning-based brain tumor classification. Brain tumors are abnormal growths of cells within the brain. Because the skull is a rigid structure, tumor growth can increase intracranial pressure and compress surrounding brain tissue, potentially causing serious neurological complications. Therefore, automated classification models can support early detection and accurate tumor categorization, contributing to clinical decision-making, treatment planning, and improved patient outcomes [39].

The performance of MCND-ComputeNet++ on the MCND eight-class dataset is compared to that on a four-class brain tumor MRI dataset in Table 9. The model performed better on the internal dataset, yielding an accuracy of 0.9738, a macro-F1 of 0.9771, a macro-AUC of 0.9993, and a macro-AP of 0.9971. The model also exhibited good generalization ability on the other dataset with an accuracy of 0.9500, macro-F1 score of 0.9490, macro-AUC of 0.9900, and macro-AP of 0.9846. But the greater ECE and Brier score on the other dataset suggest lower calibration reliability in comparison to the MCND dataset, which is likely caused by differences in class structure and dataset shift. Overall, the results demonstrate that the proposed model has a good externalization performance with respect to MRI data from a separate dataset and has a high performance and calibration level on the internal MCND data.

## 5. Discussion

Results of the experiments validate the effectiveness of the MCND-ComputeNet++ framework in the classification of brain MRI images with multiple classes. The proposed model had a mean accuracy of 0.9738, a macro-F1 score of 0.9771, and a macro-AUC score of 0.9993. The results suggest the framework is very accurate and stable in repeated partitioning of the dataset. The narrow bootstrap confidence intervals also confirm the reliability of the results obtained; a narrow interval would not occur if the performance was just a result of a nice split in the data, but would if it were a result of consistent behavior across the different subsets of data.

Additional insight into the diagnostic behavior of the model is gained from the class-wise results. The precision, recall, and F1-score values of the categories of the brain tumor (BT_glioma, BT_meningioma, and BT_pituitary) were nearly perfect, suggesting that the model was able to identify strong tumor-related MRI patterns. Analogously, good performance was obtained for Alzheimer’s disease classes, especially for the AD_MildDemented and AD_ModerateDemented classes, demonstrating the potential value of employing a multi-scale and attention-guided design for learning differences in the anatomical structure of Alzheimer’s disease. The confusion matrix, however, revealed that the errors that were made were mostly from AD_VeryMildDemented to normal and, to a lesser extent, from MS to normal. This pattern is clinically sensible, as very mild Alzheimer’s disease can have some subtle anatomical changes similar to aging, and multiple sclerosis can be heterogeneous depending on the visibility of the lesions and slice selection. The remaining errors are therefore focused mainly on the most subtle and most difficult-to-spot categories from a clinical point of view.

The ROC analysis and precision–recall analysis also show the discriminative power of MCND-ComputeNet++. All the one versus rest ROC curves were clustered in the upper left corner, with AUC values ranging between ∼0.996 and 1.000 across different classes, indicating good discrimination among eight classes. Similarly, the precision–recall curves were shifted near the upper-right corner, and the average precision score was around 0.990–1.000. The results are valuable for multi-class medical image classification, as they demonstrate that the model not only assigns high confidence to the correct class but also ranks the true class with high confidence. This can be useful when assessing models in class imbalance scenarios, when accuracy might not be the most appropriate metric.

The baseline comparison helps to support the effectiveness of the proposed architecture more. The overall performance of the MCND-ComputeNet++ model was the best, with an accuracy of 0.9738, macro-AUC of 0.9993, macro-AP of 0.9971, ECE of 0.0154, and Brier score of 0.0400. These values are superior to most commonly used pretrained models such as ResNet50, DenseNet121, EfficientNetB0, EfficientNetV2-S (with ordinary classifier), ConvNeXt-Tiny, and Swin-Tiny. Though the proposed model achieved a macro-F1 score of 0.9771, which was slightly better than ConvNeXt-Tiny with 0.9615, MCND-ComputeNet++ achieved a better accuracy, macro-AUC, macro-AP, ECE, and Brier score. This suggests that the proposed framework achieves a more balanced performance considering both accuracy in classification and reliability of confidence.

Finally, it is worth noting that EfficientNetV2-S, a standard classifier, is used as the backbone of MCND-ComputeNet++. The EfficientNetV2-S standard classifier achieved an accuracy of 0.9308, macro-F1 of 0.9500, macro-AUC of 0.9981, macro-AP of 0.9921, ECE of 0.0326, and Brier score of 0.1045. The results of the proposed model, however, showed a high accuracy of 0.9482 and a low Brier score of 0.0817. This validates that the performance boost is not just attributable to the fact that the model uses a strong backbone, EfficientNetV2-S. On the contrary, the proposed architectural components, such as multi-scale feature projection, spatial alignment, gated multi-scale fusion, convolutional refinement, and spatial attention pooling, lead to the improvement. The components enable the model to make better use of the hierarchical information compared to a standard backbone followed by a standard classifier head.

The rest of the baselines are not as good, which is also an indicator of the necessity of the proposed design. The accuracy of DenseNet121 is 0.8808, EfficientNetB0 is 0.8689, Swin-Tiny is 0.8579, and ResNet50 is 0.7720. The models that we used are standard available architectures that have already been trained, but the low scores indicate that conventional transfer learning is not enough for this multi-class MRI classification task with high heterogeneity. Generally, ResNet50 showed the lowest classification accuracy and lower probability reliability, as reflected in its Brier score (0.3120). These results show the effectiveness of MCND-ComputeNet++ for this problem, as it represents backbone features with multi-scale adaptive fusion and attention aggregation instead of only using the final backbone global feature vector.

The reliability of the proposed model is also ensured by calibration results. In the cross-validation experiment, mean ECE and mean Brier scores were 0.0293 and 0.0451, respectively, suggesting that the predicted probabilities were reasonably well-calibrated. The proposed model also performed the best in terms of ECE and Brier score in the baseline comparison with the other models compared. For medical decision-support systems, the goal is not only to classify the class correctly but also to have confidence scores that reflect the likelihood of correctness: this is important. Reliable confidence estimation can aid the clinician in assessing whether or not its predictions are reliable and whether further review is warranted.

The proposed model is also of significance with respect to the complexity of the tasks. Previous studies have reported very good performance in specific applications, such as brain tumor classification or Alzheimer’s diagnosis. But such studies tend to have a more limited range of labels and a more uniform grouping of disorders. On the other hand, the present study is performed on a more diverse set of subjects comprising Alzheimer’s disease stages, brain tumor types, multiple sclerosis (MS), and normal controls (NCs). Hence, while caution must be used when comparing the results directly to other studies because of differences in datasets, class definitions, and evaluation protocols, the results indicate that MCND-ComputeNet++ is competitive in a wider and more heterogeneous image-level MRI classification task.

Finally, the ablation and statistical analyses further enhance the validity of the proposed method. The ablation variants assess the impacts of omitting gated fusion, attention pooling, EMA, MixUp/CutMix, and calibration. Such comparisons provide additional confidence that the proposed framework is not simply a replacement for the backbone but a full computational pipeline that enhances the discriminatory, generalizational, and confidence reliability. Additional tests are conducted to determine if the differences observed in performance are statistically significant: the McNemar test and the bootstrap test. In summary, high classification scores, excellent baseline classification and validation results, and improved calibration support MCND-ComputeNet++ as a promising image-level classification framework for the classification of brain MR images into eight classes.

Comparative results in Table 10 indicate that earlier state-of-the-art studies have tended to report very high performance, although most of them were designed to operate in disease-specific scenarios, in particular, brain tumor classification or Alzheimer’s disease recognition [15,16,17,18,19,20,21,25,26,34]. Such more restricted tasks are inherently less heterogeneous than the current problem, which is a combination of eight neurological categories with one protocol. This is why direct numerical comparison is to be viewed with caution. Nevertheless, the suggested MCND-ComputeNet++ attains much more competitive results, boasting 97.38% accuracy, 97.71% macro-F1, and close-to-perfect discrimination metrics, as well as covering a significantly larger label space and incorporating components that are reliability-focused: calibration analysis, bootstrap confidence intervals, statistical testing, and Grad-CAM. This implies that the suggested framework is not limited to benchmark-level classification but provides a more detailed solution to heterogeneous neurological MRI analysis.

Another significant drawback is dataset bias. The MCND dataset may not adequately capture the differences in scanner vendor, magnetic field strength, acquisition protocols, demographics, disease severity, or annotation quality across hospitals. If the model is trained on one dataset and then tested on a different one, it could be learning the visual regularities of the particular dataset, which might not apply to another institution. This is particularly important for MRI, where there may be variations in contrast, resolution, slice thickness, and preprocessing techniques among scanners. The framework should be validated at multiple centers and tested in different domain shifts before it can be trusted for actual clinical use.

## 6. Conclusions

A deep learning framework that classifies brain MRI into multiple classes (MCND-ComputeNet++) was presented in this paper. The proposed framework has an eight-class setting that includes three Alzheimer’s disease class categories, three brain tumor class categories, multiple sclerosis, and a normal image, which will enable more detailed research on a mixture of neurological diseases than other previous studies that were limited to one type of neurological disease group. It simply generates a series of hierarchical feature maps using a single pretrained EfficientNetV2-S model backbone. They are then projected into a common latent space and then processed in a multi-scale, spatially aligned, gated way with fusion, convolution, and spatial attention, and then aggregated and sent to an eight-class classification head. There are also strategies that are used to ensure the robustness and reliability of the framework, such as class-balanced focal loss, MixUp/CutMix, exponential moving average weight smoothing, temperature scaling, and test-time augmentation.

Experiments revealed that MCND-ComputeNet++ yielded good results and stable classification results. The proposed framework was statistically reliable and obtained a small bootstrap confidence interval after undergoing five-fold cross-validation, with a high mean accuracy of 0.9738, a mean macro-F1 score of 0.9771, a mean macro-AUC of 0.9993, and a mean average precision of 0.9971. The class-wise analysis revealed that the model achieved good performance on categories of brain tumor and stages of Alzheimer’s disease, with the rest of the errors being in visually subtle classes like AD Very Mild Demented, MS, and normal. These results indicate that the model preserves prominent MRI patterns of the disease and struggles with categories with mild and heterogeneous anatomical changes.

The results obtained from the baseline comparison also showed the effectiveness of the proposed architecture. MCND-ComputeNet++ achieved the highest overall performance with an accuracy of 0.9738, macro-AUC of 0.9993, macro-AP of 0.9971, ECE of 0.0154, and Brier of 0.0400. Although the macro-F1 score of ConvNeXt-Tiny was slightly higher, it did not significantly affect the relatively good accuracy and class separability, as well as the average precision and calibration performance, of the proposed model. Most importantly, MCND-ComputeNet++ outperformed EfficientNetV2-S, a standard classification network, with the same backbone. This demonstrates that the above-mentioned selection of the backbone is not the only thing that results in the improvement; the proposed multi-scale projection, spatial alignment, gated fusion, convolutional refinement, and spatial attention pooling modules also do.

The framework was also found to be of practical use in reliability analysis. The low ECE and Brier scores indicate the ability of MCND-ComputeNet++ to give more reliable estimates of probability than the baselines tested. This is relevant for medical decision-support applications, for which the certainty of a prediction may be as crucial as the predicted label. Moreover, the proposed components are found to contribute significantly to the overall performance from the ablation and statistical studies. Both the McNemar test and the bootstrap comparison were used to verify that the difference in performance was both statistically and numerically significant.

While the findings were positive, there are some points to be noted. First, this study employed 2D MRI slices with no attempt made to exploit the inter-slice spatial continuity and volumetric anatomical context. Second, the MCND dataset is rather large and heterogeneous, but may not fully represent the diversity of scanner type, acquisition protocol, imaging center, and patient demographics in the clinical setting. Thirdly, only imaging data are currently used, and the results are not based on any other clinical information, such as demographic data, cognitive tests, radiological reports, PET imaging data, diffusion imaging data, or structured medical records. Lastly, while the calibration results are promising, the use of the results in clinical decision support must be validated with external, multi-center, and prospective datasets.

The extension of MCND-ComputeNet++ in a couple of directions is suggested for future work. The next step is to create a 3D volumetric version of the framework to better represent the 3D relationships between slices of MRI data. Joint use of multimodal neuroimaging and clinical metadata for improving diagnostic accuracy, confidence estimation, and clinical interpretability is another direction of significant interest. More advanced methods for uncertainty estimation and more powerful external validation methods are also warranted for further investigation. Thirdly, the framework needs to be validated across a range of centers to test its applicability, validity and clinical relevance at a large-scale level.

## Figures and Tables

**Table 1:** Hyperparameter configuration used for the MCND-ComputeNet++ model.

Parameter	Value
Random seed	42
Input image size	224×224
Batch size	24
Number of workers	2
Cross-validation folds	5
Maximum epochs	35
Warmup epochs	3
Early stopping patience	8
Dropout	0.30
Initial learning rate	$2\times 10^{-4}$
Minimum learning rate	$1\times 10^{-6}$
Weight decay	$1\times 10^{-4}$

**Table 2:** Fold metrics.

Metric	Fold 0	Fold 1	Fold 2	Fold 3	Fold 4
accuracy	0.9729	0.9687	0.9769	0.9765	0.9741
precision_macro	0.9739	0.9612	0.9759	0.9784	0.9589
recall_macro	0.9832	0.9831	0.9869	0.9863	0.9860
f1_macro	0.9784	0.9715	0.9817	0.9822	0.9719
precision_micro	0.9724	0.9689	0.9769	0.9769	0.9738
recall_micro	0.9724	0.9689	0.9769	0.9769	0.9738
f1_micro	0.9729	0.9687	0.9769	0.9765	0.9741
precision_weighted	0.9728	0.9702	0.9774	0.9773	0.9745
recall_weighted	0.9724	0.9689	0.9769	0.9769	0.9738
f1_weighted	0.9729	0.9688	0.9769	0.9766	0.9739
auc_macro_ovr	0.9993	0.9992	0.9994	0.9994	0.9993
auc_micro_ovr	0.9992	0.9985	0.9986	0.9993	0.9989
ap_macro	0.9975	0.9974	0.9969	0.9974	0.9962
ap_micro	0.9953	0.9912	0.9915	0.9954	0.9941
ece	0.0154	0.0118	0.0190	0.0136	0.0172
brier	0.0400	0.0320	0.0480	0.0360	0.0440
accuracy_ci	(0.9662, 0.9774)	(0.9622, 0.9744)	(0.9713, 0.9820)	(0.9713, 0.9817)	(0.9686, 0.9790)
f1_macro_ci	(0.9739, 0.9829)	(0.9580, 0.9802)	(0.9773, 0.9857)	(0.9781, 0.9862)	(0.9548, 0.9827)
auc_macro_ci	(0.9990, 0.9996)	(0.9988, 0.9995)	(0.9983, 0.9994)	(0.9991, 0.9997)	(0.9985, 0.9995)
ap_macro_ci	(0.9954, 0.9982)	(0.9940, 0.9975)	(0.9919, 0.9974)	(0.9961, 0.9986)	(0.9944, 0.9978)

Fold-level results were obtained using the EMA-weighted checkpoint selected according to the best validation macro-F1 score within each fold. Final inference used test-time augmentation (TTA), and calibration metrics, including ECE and Brier score, were computed after temperature scaling of the predicted probabilities.

**Table 3:** Classification report from cross-validation results.

Class	Precision	Recall	F1-Score
AD_MildDemented	0.9824	0.9989	0.9906
AD_ModerateDemented	0.9552	1.0000	0.9771
AD_VeryMildDemented	0.9178	0.9871	0.9512
BT_glioma	0.9920	0.9957	0.9938
BT_meningioma	0.9850	0.9895	0.9873
BT_pituitary	0.9914	0.9937	0.9925
MS	0.9474	0.9616	0.9544
Normal	0.9865	0.9544	0.9702
Accuracy	–	–	0.9738
Macro-average	0.9697	0.9851	0.9771
Weighted average	0.9744	0.9738	0.9738

**Table 4:** CV summary table.

Metric	Mean	Std	Min	Max
accuracy	0.9738	0.0033	0.9687	0.9769
precision_macro	0.9697	0.0089	0.9589	0.9784
recall_macro	0.9851	0.0018	0.9831	0.9869
f1_macro	0.9771	0.0052	0.9715	0.9822
precision_micro	0.9738	0.0034	0.9689	0.9769
recall_micro	0.9738	0.0034	0.9689	0.9769
f1_micro	0.9738	0.0033	0.9687	0.9769
precision_weighted	0.9744	0.0030	0.9702	0.9774
recall_weighted	0.9738	0.0034	0.9689	0.9769
f1_weighted	0.9738	0.0033	0.9688	0.9769
auc_macro_ovr	0.9993	0.0001	0.9992	0.9994
auc_micro_ovr	0.9989	0.0003	0.9985	0.9993
ap_macro	0.9971	0.0005	0.9962	0.9975
ap_micro	0.9935	0.0020	0.9912	0.9954
ece	0.0154	0.0028	0.0118	0.0190
brier	0.0400	0.0063	0.0320	0.0480

**Table 5:** Bootstrap confidence intervals of the proposed model.

Metric	95% Confidence Interval
Accuracy	(0.9711, 0.9760)
Macro-F1	(0.9727, 0.9807)
Macro-AUC	(0.9990, 0.9994)
Macro-AP	(0.9952, 0.9971)

**Table 6:** Ablation study summary.

Metric	Full_Model	No_Mixing	No_Calibration	No_Gated_Fusion	No_Ema	No_Attn_Pool
accuracy	0.9738	0.9476	0.9448	0.9442	0.9436	0.9375
f1_macro	0.9771	0.9633	0.9434	0.9598	0.9624	0.9399
f1_micro	0.9738	0.9476	0.9448	0.9442	0.9436	0.9370
auc_macro_ovr	0.9993	0.9980	0.9979	0.9982	0.9980	0.9983
ap_macro	0.9971	0.9909	0.9921	0.9924	0.9917	0.9935
ece	0.0154	0.0431	0.2213	0.0432	0.0458	0.0411
brier	0.0400	0.0840	0.1556	0.0899	0.0948	0.0988
accuracy_ci_low	0.9686	0.9409	0.9363	0.9372	0.9363	0.9297
accuracy_ci_high	0.9793	0.9547	0.9526	0.9521	0.9517	0.9450
f1_macro_ci_low	0.9747	0.9577	0.9192	0.9546	0.9573	0.9196
f1_macro_ci_high	0.9833	0.9682	0.9591	0.9660	0.9678	0.9550

For the ablation study, the full model was evaluated using EMA weights, TTA, and temperature scaling. In each ablation variant, only the indicated component was removed, while the remaining training, inference, and model-selection settings were kept fixed. For the no_calibration variant, temperature scaling was not applied; therefore, ECE and Brier score were computed from the uncalibrated probabilities.

**Table 7:** Statistical tests versus the full model.

Metric	No_Mixing	No_Attn_Pool	No_Ema	No_Gated_Fusion	No_Calibration
mcnemar_b	36	99	78	84	73
mcnemar_c	122	66	65	73	64
chi2	45.7278	6.2061	1.0070	0.6369	0.4672
*p*_value	1.36 $\times 10^{-11}$	1.27 $\times 10^{-2}$	3.16 $\times 10^{-1}$	4.25 $\times 10^{-1}$	4.94 $\times 10^{-1}$
delta_accuracy_mean	−0.0262	−0.0363	−0.0302	−0.0296	−0.0290
delta_accuracy_ci_low	−0.0384	−0.0496	−0.0430	−0.0421	−0.0430
delta_accuracy_ci_high	−0.0139	−0.0229	−0.0169	−0.0165	−0.0160
delta_f1_macro_mean	−0.0138	−0.0337	−0.0173	−0.0147	−0.0372
delta_f1_macro_ci_low	−0.0256	−0.0638	−0.0260	−0.0288	−0.0641
delta_f1_macro_ci_high	−0.0064	−0.0197	−0.0069	−0.0087	−0.0155

**Table 8:** Baseline comparison between the proposed MCND-ComputeNet++ and standard pretrained models for multi-class brain MRI classification.

Model	Accuracy	Macro-F1	Macro-AUC	Macro-AP	ECE	Brier
Proposed MCND-ComputeNet++	0.9738	0.9771	0.9993	0.9971	0.0154	0.0400
ConvNeXt-Tiny	0.9421	0.9615	0.9980	0.9863	0.0367	0.0904
EfficientNetV2-S standard classifier	0.9308	0.9500	0.9981	0.9921	0.0326	0.1045
DenseNet121	0.8808	0.9218	0.9964	0.9851	0.0330	0.1680
EfficientNetB0	0.8689	0.8856	0.9951	0.9803	0.0400	0.1808
Swin-Tiny	0.8579	0.8982	0.9925	0.9619	0.0334	0.1971
ResNet50	0.7720	0.8329	0.9862	0.9409	0.0389	0.3120

All baseline models and the proposed MCND-ComputeNet++ were evaluated using the same data split, preprocessing pipeline, training schedule, and model-selection criterion. The final checkpoint was selected based on the best validation macro-F1 score. Unless otherwise stated, final inference used EMA weights, TTA, and temperature scaling before reporting ECE and Brier score.

**Table 9:** Dataset comparison.

Dataset	Num. Classes	Train Size	Val Size	Test Size	Accuracy	Macro-F1	Macro-AUC	Macro-AP	ECE	Brier
MCND	8	11,480	1640	3280	0.9738	0.9771	0.9993	0.9971	0.0154	0.0400
Brain Tumor MRI	4	4760	840	1600	0.9500	0.9490	0.9900	0.9846	0.0431	0.0876

Note: The results were computed using the final selected checkpoint based on validation macro-F1. Final inference used EMA weights, TTA, and temperature scaling. The same preprocessing and inference protocol was applied to the external dataset to assess generalization under a consistent evaluation setting.

**Table 10:** Comparative analysis between the proposed framework and representative previous studies.

Ref.	Study	Main Task/Setting	Reported Performance
[15]	Islam et al., 2023	Brain tumor MRI classification	Weighted accuracy 98.73%, weighted F1 95.29%
[16]	Priyadarshini et al., 2024	Multigrade brain tumor MRI classification	Accuracy 96.20%
[17]	Jain et al., 2023	Transfer learning comparison for brain tumor MRI	VGG16 96.00%, ResNet50 89.00%, InceptionV3 75.00% accuracy
[18]	Ishaq et al., 2025	Multigrade brain tumor detection and classification	Accuracy 98.60%
[19]	Vinukonda et al., 2025	Alzheimer’s MRI multi-class classification	Accuracy 98.12%, highest class AUC 0.97
[21]	Sorour et al., 2024	MRI-based Alzheimer’s classification	Accuracy 95.17% for 3D, 93.61% for 2D; CNN-LSTM up to 99.92% in one configuration
[25]	Hashemi et al., 2022	MS lesion segmentation	DSC 82.30%
[26]	Huang et al., 2024	Joint MS/NMOSD segmentation–classification	DSC 74.87%, classification accuracy 92.36%
[34]	Srinivas et al., 2026	Explainable brain tumor MRI classification	Accuracy 95.86%, validation accuracy 95.32%, precision/recall/F1 ≈ 0.95
Proposed model	MCND-ComputeNet++	Eight-class heterogeneous neurological MRI classification	Accuracy 97.38%, macro-F1 97.71%, macro-AUC 99.93%, macro-AP 99.71%

## Data Availability

The data presented in this study are openly available in https://www.kaggle.com/datasets/alifatahi/multi-class-neurological-disorder-mcnd-dataset (accessed on 1 April 2026) and https://www.kaggle.com/datasets/masoudnickparvar/brain-tumor-mri-dataset (accessed on 4 May 2026).
